# MicroRNAs Regulating Hippo-YAP Signaling in Liver Cancer

**DOI:** 10.3390/biomedicines9040347

**Published:** 2021-03-30

**Authors:** Na-Hyun Lee, So Jung Kim, Jeongeun Hyun

**Affiliations:** 1 Institute of Tissue Regeneration Engineering (ITREN), Dankook University, Cheonan 31116, Korea; nhlee0609@dankook.ac.kr (N.-H.L.); r00by8340@dankook.ac.kr (S.J.K.); 2 Department of Nanobiomedical Science and BK21 PLUS NBM Global Research Center for Regenerative Medicine, Dankook University, Cheonan 31116, Korea; 3 Department of Regenerative Dental Medicine, College of Dentistry, Dankook University, Cheonan 31116, Korea

**Keywords:** microRNAs, hepatocellular carcinoma, Hippo kinase, Yes-associated protein, liver disease, diagnosis, prognosis, cancer therapy

## Abstract

Liver cancer is one of the most common cancers worldwide, and its prevalence and mortality rate are increasing due to the lack of biomarkers and effective treatments. The Hippo signaling pathway has long been known to control liver size, and genetic depletion of Hippo kinases leads to liver cancer in mice through activation of the downstream effectors yes-associated protein (YAP) and transcriptional coactivator with PDZ-binding motif (TAZ). Both YAP and TAZ not only reprogram tumor cells but also alter the tumor microenvironment to exert carcinogenic effects. Therefore, understanding the mechanisms of YAP/TAZ-mediated liver tumorigenesis will help overcome liver cancer. For decades, small noncoding RNAs, microRNAs (miRNAs), have been reported to play critical roles in the pathogenesis of many cancers, including liver cancer. However, the interactions between miRNAs and Hippo-YAP/TAZ signaling in the liver are still largely unknown. Here, we review miRNAs that influence the proliferation, migration and apoptosis of tumor cells by modulating Hippo-YAP/TAZ signaling during hepatic tumorigenesis. Previous findings suggest that these miRNAs are potential biomarkers and therapeutic targets for the diagnosis, prognosis, and treatment of liver cancer.

## 1. Introduction

Liver cancer is one of the leading causes of cancer-related death worldwide, with a 5-year survival rate as low as 30–40% [1,2]. Hepatocellular carcinoma (HCC) is the most common type of primary liver cancer and occurs in patients with chronic liver disease [3]. Despite remarkable advances in research to overcome viral hepatitis, which is the biggest cause of HCC, the incidence and mortality of liver cancer are still increasing along with the increased prevalence of nonviral steatohepatitis. However, the complex pathophysiology of liver cancer has limited the development of effective diagnosis and therapeutic intervention, prompting a comprehensive understanding of liver carcinogenesis.

Many studies have revealed that several developmental pathways, such as Wnt/β-catenin, Hedgehog (Hh) and Hippo/Yes-associated protein (YAP) signaling, contribute to hepatic carcinogenesis [4]. For example, Hh signaling is inactive in the normal liver of adult mice and humans but reactivated in chronic liver diseases and liver cancers, promoting liver fibrosis and hyperplasia and constructing a tumor-favorable microenvironment [5]. Likewise, overexpression of YAP in mice massively increases the size of the liver due to hyperproliferation of liver cells and ultimately promotes hepatic tumorigenesis [6]. The hepatocyte-specific induction of YAP nuclear localization dedifferentiates hepatocytes into ductal-like progenitor cells that are highly proliferative [7]. Similar results have been found after genetic ablation of one of the key components of the Hippo signaling pathway, including Neurofibromin 2 (Nf2), Mammalian STE20-like 1/2 (Mst1/2), and Large Tumor Suppressor 1/2 (Lats1/2), which regulate the activity of YAP and transcriptional coactivator with PDZ-binding motif (TAZ), another downstream effector of Hippo signaling, by phosphorylation and subsequent proteasomal degradation [8,9]. For example, Nf2 conditional null mice show hyperproliferation of hepatic progenitor cells, known as a ductular reaction, and develop both HCC and intrahepatic cholangiocarcinoma (ICC) [10,11]. Notably, codeletion of Yap suppresses liver overgrowth, progenitor expansion and tumor development in Nf2 knockout mice, demonstrating that Yap drives hepatic tumorigenesis caused by inactivated Hippo signaling [10]. Accordingly, it has been observed that the expression of NF2 negatively correlates with the expression of YAP in liver tissues of patients with HCC or ICC [12,13]. Except for NF2, for which missense mutations have been discovered in 1.9% and 5.3% of human HCC and ICC cases, respectively [12], somatic or germline mutations in neither MST1/2 nor LATS1/2 genes have been reported in common cancers [14]. This suggests that post-transcriptional regulation or epigenetic silencing, rather than DNA mutations, of the Hippo signaling pathway may play central roles in the aberrant inactivation of Hippo signaling and hyperactivation of YAP/TAZ transcriptional coactivators in liver cancers. Nevertheless, whether and how the activity of the Hippo and YAP/TAZ signaling pathways are regulated post-transcriptionally has not been well investigated.

MicroRNAs (miRNAs) are a group of small noncoding RNAs that are approximately 21–25 nucleotides in length [15]. miRNAs regulate the expression of approximately 30% of protein-coding genes at the post-transcriptional level by binding directly to target messenger RNA (mRNA), which results in translational suppression or degradation of target mRNAs [16]. Many studies have demonstrated that miRNAs play important roles in diverse biological processes, including cell proliferation, differentiation, and death; thus, abnormal regulation of miRNAs can lead to various pathological conditions, including liver cancers [17]. miRNAs have become promising candidates for biomarkers and therapeutic targets in many cancers due to their regulatory functions and detectable properties in various biological fluids, including blood, saliva, and urine.

Recently, several miRNAs have been reported to be associated with Hippo-YAP/TAZ signaling in the control of liver cancer cell behaviors [18,19]. Here, we introduce recent findings of the interaction between miRNAs and Hippo-YAP/TAZ signaling components in the development and progression of liver cancers. We also discuss what remains to be addressed in future studies to improve our knowledge of the underlying mechanisms whereby dysregulation of miRNA and Hippo-YAP/TAZ signaling contributes to hepatic malignancies. Finally, we propose the potential of these miRNAs in clinical applications for the diagnosis, prognosis, and treatment of liver cancers.

## 2. Hippo-YAP/TAZ Signaling Pathway

**Figure 1:**
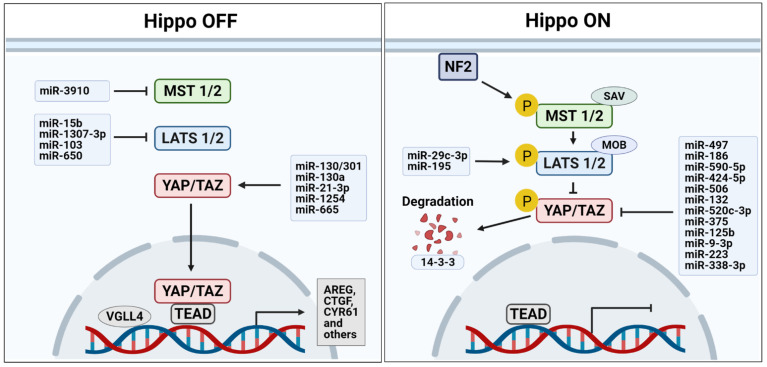
MicroRNAs that regulate the core components of Hippo signaling pathway. Several microRNAs are involved in kinase cascade (MST1/2 and LATS1/2) and downstream effectors (YAP/TAZ) of the Hippo signaling pathway in liver cancer as either oncogenes or tumor suppressors. Abbreviations: NF2, Neurofibromin 2; MST1/2, Mammalian STE20-like 1/2; SAV, Salvador; LATS1/2, Large Tumor Suppressor 1/2; MOB, Mps one binder kinase activator; YAP/TAZ, Yes-associated protein and transcriptional coactivator with PDZ-binding motif; TEAD, TEA domain family member; VGLL4, Vestigial like family member 4; AREG, amphiregulin; CTGF, connective tissue growth factor; CYR61, Cysteine-rich angiogenic inducer 61.

Hippo signaling, which was first discovered in *Drosophila*, is evolutionarily conserved in vertebrates as a key regulator of organ growth [20,21]. In mammals, two serine/threonine kinases, the STE20-like protein kinase MST1/2 (Hpo in *Drosophila*) and the NDR family protein kinase LATS1/2 (Wts in *Drosophila*), consist of a core kinase cascade of the Hippo signaling pathway [8,22] (Figure 1). The MST1/2 kinases form a complex with Salvador 1 (SAV1, Sav in *Drosophila*) to phosphorylate and activate LATS1/2 kinases, which in turn phosphorylate and sequester the two major downstream effectors of the Hippo pathway, YAP and TAZ (Yki in *Drosophila*), by promoting the association of YAP/TAZ with 14-3-3 proteins in the cytoplasm where ubiquitin-mediated degradation of YAP and TAZ occurs [8,23,24,25]. MST1/2 also interact with Mps one binder kinase activator-like 1A/B (MOBKL1A/B, Mats in *Drosophila*), which enhances the activity of LATS1/2 kinases [26,27]. When Hippo signaling activity is suppressed, YAP and TAZ are dephosphorylated, translocate into the nucleus, and function as transcriptional coactivators via interactions with various transcription factors, such as p73, TEA domain family member (TEAD, Sd in *Drosophila*), SMAD, and Runt-related transcription factor (RUNX), to induce the expression of genes promoting cell proliferation and inhibiting apoptosis [25]. For example, *AREG* [28], *BIRC5* [29], *CCNE1* [30], *CTGF* [31,32], *CYR61* [30,32], and *GLI2* [33] have been identified as direct target genes of both YAP and TAZ. The upstream regulators of the Hippo kinase cascade include FAT1-4 (Fat in *Drosophila*), Merlin (encoded by the *NF2* gene; Mer in *Drosophila*), KIBRA (Kibra in *Drosophila*), RASSF, and Ajuba [25]. MST1/2 kinases are activated by FAT1-4 through the apical protein FRMD6/Willin (Ex in *Drosophila*) that forms a complex with two other apically localized proteins, Merlin and KIBRA [34,35]. In contrast, both RASSF and Ajuba inhibit the Hippo signaling pathway by competing with SAV1 for binding with MST1/2 [36]. In addition, YAP and TAZ sense extracellular mechanical stimuli, such as extracellular matrix (ECM) stiffness and cell geometry, and integrate and convert them into intracellular molecular signals, resulting in changes in cellular behaviors, including cell proliferation, migration, and transdifferentiation [37]. 

## 3. The Roles of the Hippo-Yap/Taz Signaling Pathway in Hepatic Tumorigenesis

Hippo-YAP/TAZ signaling is well known to control organ size during development and to mediate the expansion of tissue-specific progenitor cells during tissue regeneration and normal cell proliferation [38]. Accumulated evidence shows aberrant expression of Hippo kinases, YAP/TAZ and their partners in many human cancers, including liver cancers [39,40,41]. In normal livers, Hippo kinases act as tumor suppressors by inhibiting hepatocyte proliferation and maintaining the differentiated state of hepatocytes [42]. In contrast, loss of Hippo kinase activities, as in mice with a genetic deletion of Nf2 [10,43], Mst1/2 [43,44], Lats1/2 [45,46], or Sav1 [47], causes hepatomegaly and liver cancers, including HCC, ICC, and/or the HCC/ICC mixed form. YAP, on the other hand, functions as an oncogene [48]. Overexpression of YAP phenocopies Hippo signaling deficiency in mice, as shown by liver overgrowth, which is mediated by an increase in hepatocyte proliferation coordinated with a decrease in hepatocyte death [49]. Moreover, removal of Yap in mice with Hippo signaling components knocked out prevented hepatomegaly and hepatic tumor development [10], indicating that YAP is required for hyperplastic cell proliferation and oncogenic transformation of liver cells. It has also been revealed that YAP induces the epithelial-to-mesenchymal transition (EMT), suppression of apoptosis, growth factor-independent proliferation, and anchorage-independent growth of cancer cells, which are attributes of cancer stem cells that are responsible for the major causes of cancer mortality, such as chemoresistance, metastasis, and recurrence [40].

Recent studies have demonstrated that activation of YAP and TAZ in tumor cells also fosters a tumor-favorable microenvironment by communicating with neighboring stromal cells [50,51]. For example, activated YAP/TAZ in hepatocyte-specific Mst1/2 knockout mice creates an inflammatory tumor microenvironment by increasing the production of inflammatory cytokines to suppress immune clearance of transformed hepatocytes by recruiting tumor-associated macrophages and to promote liver cancer development [38,41]. In addition, both tumor cells and cancer-associated fibroblasts increase the stiffness of extracellular matrices, which activates YAP/TAZ mechanosensors to mediate the metabolic crosstalk between tumor cells and cancer-associated fibroblasts, providing them with sufficient nutrients for tumor growth and maintenance of a cancer-prone microenvironment [52,53,54]. Hence, Hippo-YAP/TAZ signaling has been targeted for developing anticancer therapeutics [21].

Most Hippo-YAP/TAZ signaling genes are rarely mutated in liver cancers, which strongly suggests that molecular events, such as epigenetic or post-transcriptional regulation in response to mechanical stresses from the tumor microenvironment, other than DNA mutations, may cause dysregulation of the Hippo-YAP/TAZ signaling pathway in in liver cancers [19,40]. 

## 4. MiRNAs Interacting with the Hippo-Yap/Taz Signaling Pathway in Liver Cancer

Dysregulation of biogenesis and expression of miRNAs affect the incidence and progression of liver cancers [55]. miRNAs can function as either tumor suppressors or oncogenes depending on their target mRNAs and resultant phenotypic changes in the cells [56]. In this section, we discuss the interplay between miRNAs and the components of the Hippo-YAP/TAZ signaling pathway that play important roles in hepatic cancer biology (Figure 1).

### 4.1. MiRNAs as Tumor Suppressors

#### 4.1.1. MiRNAs Targeting YAP or TAZ

Bioinformatic algorithms performed by Liu et al. [57] identified that miR-375 binds directly to the 3′ untranslated region of YAP mRNA. In PLC/PRF/5 and MHCC-97L HCC cell lines, ectopic expression of miR-375 downregulated the expression level of YAP protein and CTGF mRNA, which can be transcribed by activated YAP [57]. Recently, Dinh and Jewell et al. [58] found that miR-375 is the most downregulated miRNA in primary fibrolamellar carcinoma (pFLC), a rare liver cancer that primarily affects adolescents and young adults, compared with nonmalignant human livers. The loss of miR-375 was induced by the presence of the DNAJB1-PRKACA fusion gene, a hallmark of FLC, although it is yet unknown how DNAJB1-PRKACA inhibits miR-375 expression [58]. As in HCC cells, the overexpression of miR-375 in FLC cells inhibited YAP and CTGF, mitigating the proliferative and migratory ability of tumor cells [58]. Another miRNA, miR-186 [59], also decreases the expression of YAP and CTGF by directly disrupting YAP mRNA, inhibiting the proliferation, migration, and invasion of HepG2, Hep3B and SNU398 HCC cell lines [59]. CTGF plays a central role in tissue remodeling and liver fibrosis, which increases the risk for cancer development and progression [60]. Moreover, CTGF is associated with tumor progression by forcing crosstalk between cancer cells and hepatic stellate cells to form a tumor-favorable microenvironment [60]. Thus, the miRNAs discussed above might have tumor suppressive effects not only by killing cancer cells but also by targeting the tumor microenvironment (Table 1). 

Both miR-506 and miR-132 also have binding sites in the 3′ untranslated region of YAP mRNA [61,62]. It was shown that the expression of miR-506 is significantly reduced in human HCC tissues and inversely correlates with the expression of the YAP gene [61]. It was experimentally revealed that miR-506 inhibits the growth of HepG2 and H7402 HCC cell lines by downregulating YAP expression [61]. miR-132 promotes apoptosis and suppresses proliferation and invasion of Huh-7 and HepG2 HCC cell lines through direct inhibition of YAP [62]. When miR-132 was cotransfected with miR-520-3p, another tumor suppressive miRNA modulating GPC3, into the Huh-7 HCC cell line, the antiproliferative and proapoptotic functions of both miRNAs on the HCC cell line were shown to be enhanced by minimizing the level of YAP [63]. Similarly, Piao et al. [64] reported that miR-424-5p decreases YAP expression, attenuates proliferation and induces the apoptosis of ICC cell lines, including CCLP-1, RBE and HuCCT-1 [64]. Zhang et al. [65] showed the in vitro anticancer effect of miR-497 by suppressing its functional target, YAP. Overexpression of miR-497 inhibits the growth and survival of HCC cell lines such as HepG2 and Huh-7, whereas silencing of miR-497 has the opposite effect [65].

Chemoresistance is one of the major issues in HCC treatment [66]. Chen et al. [66] found that the level of miR-590-5p negatively correlates with YAP expression in HCC, which is resistant to adriamycin (also known as doxorubicin), the most common first-line chemotherapeutic agent for transarterial chemoembolization [66]. Furthermore, they found that miR-590-5p directly targets YAP and that dysregulation of the miR-590-5p/YAP axis leads to chemoresistance in HCC. The potential anticancer role of miR-509-3p through the suppression of YAP in cholangiocarcinoma (CCA) has been reported by Jung et al. [67]. They found that anticancer drugs such as gemcitabine show synergistic effects when treated in combination with a novel inhibitor of histone deacetylase, CG200745, in CCA cell lines and that CG200745 effectively suppresses tumor growth in xenograft mouse models of gemcitabine-resistant CCA [67]. By performing a microarray and a miRNA array, they further found that CG200745 inhibits the expression of YAP-TEAD4 signaling and increases the expression of miR-509-3p, which downregulates the level of YAP protein [67], possibly by directly targeting YAP mRNA, as in ovarian cancer cells [68].

miRNAs that target TAZ, a paralog of YAP, in HCC include miR-338-3p, miR-9-3p, and miR-125b [69,70,71]. miR-338-3p has also been reported to be suppressed by the hepatitis B virus (HBV)-encoded protein preS2, upregulating the expression of TAZ in HBV-associated HCC [69]. Both miR-9-3p and miR-125b are known to be downregulated in human HCC tissues and hepatic tumor cells, while the expression of TAZ is upregulated [70,71]. In particular, miR-9-3p is involved in the noninvasive proliferation of tumor cells via signaling pathways, including AKT, ERK1/2, and β-catenin [70]. miR-125b is capable of inhibiting cell invasion and migration through the regulation of TAZ expression [71]. However, the roles of miR-9-3p and miR-125b in human cancer are controversial, and they could function as either tumor suppressors or oncogenes depending on the type of cancer [70,71]. For example, miR-9-3p has been reported as a tumor suppressor in liver, breast, oral, and gastric cancers, whereas it is known as an oncogene primarily in brain cancer [70]. miR-223 plays a critical role in the progression of nonalcoholic steatohepatitis (NASH) to HCC by targeting Taz and the inflammatory gene Cxcl10 in mouse hepatocytes [72]. He et al. [72] found that chronic high-fat diet treatment in miR-223 knockout mice increases the prevalence of liver cancer by activating oncogenic and inflammatory pathways through TAZ and CXCL10 compared to wild-type mice. These findings are consistent with other reports that the level of miR-223 is substantially downregulated in human HCC [72] and that chronic inflammatory injury promotes hepatobiliary carcinogenesis [72].

**Table 1:** List of tumor suppressive microRNAs inactivating YAP/TAZ and their effect(s) on liver cancer.

MiRNA Name	Direct Target(s) ^†^ (Activator or Suppressor)	Effect(s) ^‡^	Disease Type	Ref.
miR-29c-3p	DNMT3B suppressor	LATS1 methylation ↓	HCC	[73]
miR-195	LATS2 activator	Apoptosis ↑	HCC	[74]
miR-497	YAP1 suppressor	Proliferation ↓, apoptosis ↑	HCC	[65]
miR-186	YAP1 suppressor	Proliferation, migration ↓	HCC	[59]
miR-590-5p	YAP1 suppressor	Chemoresistance ↓	HCC	[66]
miR-424-5p	YAP1 suppressor	Proliferation ↑, apoptosis ↓	HCC	[64]
miR-506	YAP1 suppressor	Proliferation ↓	HCC	[61]
miR-132	YAP1 suppressor	Apoptosis ↑	HCC	[62]
miR-375	YAP1 and CTGF suppressor	Growth, invasion ↓	FLC	[58]
	YAP1/2 suppressor	Proliferation, invasion ↓	HCC	[57]
miR-125b	TAZ suppressor	Migration, invasion ↓	HCC	[71]
miR-9-3p	TAZ suppressor	Proliferation ↓	HCC	[70]
miR-223	TAZ suppressor	Neutrophil activation(proinflammatory mediators) ↓	NASH, HCC	[72]
miR-338-3p	TAZ suppressor	preS2 expression ↓	HCC (HBV derived)	[69]

Abbreviation: miR, microRNA; HCC, hepatocellular carcinoma; FLC, fibrolamellar carcinoma; NASH, nonalcoholic steatohepatitis; HBV, hepatitis B virus. ^†^ All direct targets were confirmed by luciferase reporter assay. ^‡^ ↑ indicates the promotion, ↓ indicates the suppression.

#### 4.1.2. MiRNAs Interacting with LATS1/2

Yang et al. [75] reported that miR-195 is one of a few miRNAs expressed differently between the drug-resistant HCC cell line BEL-7402/5-FU and its parental cell line BEL-7402. miR-195 was shown to be downregulated in HCC cells that acquired drug resistance [75]. Overexpression of miR-195 upregulates the expression of LATS2 and downregulates BCL-w, an anti-apoptotic protein, which sensitizes BEL-7402/5-FU cells to anticancer drugs by suppressing proliferation and inducing apoptosis of the cells [74,75].

Some miRNAs play a tumor suppressive role by interacting with other epigenetic regulators [76]. For example, Wu et al. [73] found that miR-29c-3p promotes DNA demethylation of the LATS1 gene through the direct inhibition of DNA methyltransferase 3B (DNMT3B), resulting in upregulated LATS1 expression (and thus increased Hippo kinase activity) to suppress oncogenic YAP activation. Consistent with the findings that the expression of DNMT3B is increased in many malignancies [73], Wu et al. [73] observed that the level of DNMT3B is higher in HCC tumor tissues than in adjacent nontumor tissues, while miR-29c-3p and LATS1 are expressed at lower levels in HCC tumors than in nontumors. Moreover, patients with either high DNMT3B, low LATS1 or low miR-29c-3p/LATS1 with high DNMT3B have worse outcomes [73].

### 4.2. MiRNAs as Oncogenes 

#### 4.2.1. MiRNAs Targeting MST1 and LATS1/2

Cheng et al. [77] showed that miR-3910 is highly expressed in human HCC tissues and various HCC cell lines compared to its level in nontumor liver tissues and normal liver cells. They found that miR-3910 directly inhibits the expression of MST1, promoting the growth and migration of HCC cells in vitro and tumor formation in vivo through the activation of oncogenic YAP (Table 2). 

Other studies have shown that miRNAs, including miR-103 [78] and miR-650 [79], directly bind to and inhibit LATS2 mRNA, leading to an increase in YAP, which promotes EMT, metastasis, and invasion of cancer cells, particularly in the liver. In addition, miR-1307-3p is induced by MEIS2, a homeobox protein that promotes HCC development and downregulates LATS1 [80]. Thus, the oncogenic function of MEIS2 is accomplished by the miR-1307-3p/LATS1 axis promoting YAP nuclear translocation in addition to its association with Wnt/β-catenin signaling [80]. Interestingly, a recent study reported that miR-15b in extracellular vesicles (EVs) derived from macrophages after exposure to arsenite, a carcinogen, is transferred to HCC cells and inactivates Hippo signaling by directly targeting LATS1 to promote the proliferation, migration, and invasion of HCC cells [81]. This study suggested the role of miR-15b as a messenger between tumor-associated macrophages and tumor cells in the progression of HCC and that targeting miR-15b could be a strategy for the treatment of liver cancer.

#### 4.2.2. MiRNAs Interacting with YAP/TAZ

Fibrosis is known as a precancerous condition that raises the risk for cancers, including liver cancer [82], and ECM stiffness increases in liver fibrosis and cirrhosis [83]. The mechanosensitive miRNAs of the miR-130/301 family have been shown to regulate fibrosis-related pathways, and their expression depends on the activation of YAP/TAZ, which is promoted by and enforces ECM stiffening [84]. miR-130a also plays a central role in positive feedback regulation of YAP expression by directly inhibiting VGLL4, an antagonist of YAP for TEAD binding, after its expression is induced by YAP [85]. Consequently, endogenous miR-130a strengthens the YAP-TEAD complex, which is a major player in hepatic carcinogenesis [85].

In addition, Hu et al. [86] reported that miR-665 is highly expressed in HCC, suppresses LATS1 activity and enhances activated YAP by negatively regulating the tyrosine phosphatase receptor type B (PTPRB) gene, the protein of which phosphorylates the LATS1 Hippo kinase. As a result, overexpression of miR-665 markedly enhances EMT, cell cycle progression, migration, and invasion of HepG2 cells in vitro [86]. Furthermore, Hu et al. [86] demonstrated that miR-665 promotes tumor growth and metastasis in HepG2 xenograft mouse models in vivo. Recently, Lu et al. [87] reported that miR-1254 is upregulated in human HCC tissues compared with adjacent nontumor tissues and various HCC cell lines, including Hep3B and Huh-7. They also demonstrated that miR-1254 promotes the proliferation, migration, and invasion of HCC cells in vitro and enhances tumor size, vascular invasion, histological grade of HCC assessed by the Edmondson-Steiner scoring system, and lung metastasis in xenografts in vivo [87]. Notably, they found that paired box gene 5 (PAX5) is directly regulated by miR-1254 and that miR-1254 inhibits the phosphorylation of LATS1 and YAP, which seems to be dependent on the availability of PAX5 [87]. Consequently, overexpression of miR-1254 promotes HCC progression [87].

TGF-β signaling is closely associated with YAP/TAZ signaling, and both signaling pathways are known to involve many cellular processes, including proliferation, differentiation and tumor formation and progression [88,89]. Hong et al. [90] reported that miR-21-3p is enriched in human HCC tissues and exerts oncogenic effects on hepatocarcinogenesis and HCC progression by targeting SMAD7, one of the negative regulators of the TGF-β signaling pathway [90], to upregulate oncogenic YAP.

**Table 2:** List of oncogenic microRNAs activating YAP/TAZ and their effect(s) on liver cancer.

MiRNA Name	Direct Target(s) ^†^ (Activator or Suppressor)	Effect(s) ^‡^	Disease Type	Ref
miR-3910	MST1 suppressor	YAP-TEAD ↑	HCC	[77]
miR-15b	LATS1 suppressor	M2 polarization (MΦ) ↑, proliferation, migration, invasion (tumor) ↑	HCC	[81]
miR-1307-3p	LATS1 suppressor	YAP-Wnt/β-catenin signaling ↑	HCC	[80]
miR-103	LATS2 suppressor	Metastasis, EMT ↑	HCC	[78]
miR-650	LATS2 suppressor	Metastasis, EMT ↑	HCC	[79]
miR-130/301	YAP/TAZ activator	ECM remodeling, fibrosis ↑	NASH	[84]
miR-130a	YAP-TEAD activator	VGLL4 (YAP antagonist) ↓	HCC	[85]
miR-21-3p	SMAD7 suppressor	TGF-β, YAP1 ↑	HCC	[90]
miR-1254	PAX5 suppressor	Hippo pathway ↓	HCC	[87]
miR-665	PTPRB suppressor	Hippo pathway ↓, EMT ↑	HCC	[86]

Abbreviation: miR, microRNA; MΦ, macrophage; HCC, hepatocellular carcinoma; NASH, nonalcoholic steatohepatitis. ^†^ All direct targets were confirmed by luciferase reporter assay. ^‡^ ↑ indicates the promotion, ↓ indicates the suppression.

## 5. Potential for Clinical Use of MiRNAs Interacting with the Hippo-YAP/TAZ Signaling Pathway in Liver Cancer

Liver cancer is often asymptomatic in the early stages and is usually diagnosed after the advent of metastasis and advanced stages, limiting the chances for surgical treatment with an optimistic prognosis [17]. Although liver biopsy is currently the gold standard for diagnosing liver cancer, it can cause severe complications due to its invasiveness and is restricted to some subset of patients [91]. Various blood-based tests are used clinically, but the currently available biomarkers show insufficient specificity and sensitivity [92]. Hence, there is an unmet need for biomarkers that can diagnose liver cancer early and differentiate the stage of liver cancer. Over the past several years, many studies have focused on identifying circulating miRNAs that have the potential to be used as biomarkers of liver cancer, since they can be detected in cell culture media and different biological fluids, such as serum, plasma, saliva, tears, urine, and breast milk [93,94,95]. Unlike cellular miRNAs or other RNAs that are degraded within a few seconds, circulating miRNAs are relatively stable, viable for a long time and resistant to endogenous RNase activity in the extracellular environment [96]. Chen et al. [97] reported that circulating miRNAs remain stable under harsh conditions, such as boiling, high or low pH, prolonged storage time, and multiple freeze–thaw cycles. Furthermore, circulating miRNAs in serum maintain their expression patterns after incubation for 24 h at room temperature and are still detectable in serum after a maximum of 10 freeze–thaw cycles [97].

However, no reports have determined whether the miRNAs involved in hepatic carcinogenesis that regulate Hippo-YAP/TAZ signaling can be released into the circulation under either normal or pathological conditions. However, previous findings that some of the miRNAs discussed above (e.g., miR-15b [81,98], miR-9-3p [70], miR-223 [72]) are also detected in the serum of liver cancer patients give rise to the intriguing question of whether they could predict the risk or progression of liver cancer. In lung cancer, for example, upregulation of miR-328-3p targeting NF2 to inactivate Hippo kinase activities is promoted by hypoxic bone marrow mesenchymal stem cells, which deliver EVs containing miR-328-3p to the lung tissue [99]. miR-328-3p is detected not only in cancer tissues but also in the serum of lung cancer patients, suggesting the potential of miR-328-3p as both a biomarker and a therapeutic target of lung cancer [99]. Moreover, miRNAs can monitor tissue responses to therapeutic interventions [100]. Bie et al. [101] have shown that the miRNA expression profiles changes in BEL-7402 HCC cells after treatment with baicalein, an anticancer drug. Notably, the putative target genes for the differentially expressed miRNAs after baicalein treatment are enriched in pathways involved in cell proliferation, including the Hippo signaling pathway [101], indicating that the miRNAs interacting with Hippo-YAP/TAZ signaling represent the status of liver cancer.

Small molecules, such as pazopanib, dasatinib, and statins, which are under investigation in clinical trials or used currently for the treatment of liver cancer, have been reported to activate the Hippo signaling pathway, thereby reducing cancer cell viability and sensitizing tumor cells to chemotherapeutics [102,103]. Verteporfin, a YAP inhibitor, disrupts the YAP-TEAD interaction by promoting the degradation of YAP [104]. Thus, an anticancer approach using verteporfin has been suggested for liver cancers with YAP overexpression and chemoresistance [105,106]. Nevertheless, adverse side effects of these small molecules have been reported, and toxicity, short life, and unintended outcomes also limit the use of these inhibitors [107]. miRNAs may become the alternative since miRNA-based therapy is effective and biologically safe [108,109]. The efficacy of mimics of tumor suppressive miRNAs or inhibitors of oncogenic miRNAs in the prevention and treatment of liver cancer has been evaluated in preclinical models of HCC. To improve the efficiency of miRNA delivery and targeting to specific organs, nanoparticles such as liposomes have been used as delivery vehicles [110,111]. The first-in-human phase I study of miRNA-based therapy was recently completed and used a liposomal miR-34 mimic (known as MRX34, Mirna Therapeutics, Inc.) in solid tumors, including HCC [112]. Although MRX34 was shown to regulate its target genes dose-dependently, unexpected immune-related adverse events occurred in a small subset of patients, the reason for which needs to be elucidated [112]. Hence, further studies are strongly encouraged to understand the mechanism of action of miRNA-based cancer therapeutics to develop more therapeutic candidates that can be used in clinical trials.

## 6. Conclusions

Genetic ablation of Hippo signaling and overactivation of YAP cause liver cancer in mice [41,113], but the Hippo-YAP/TAZ signaling pathway is dysregulated in human liver cancer primarily by molecular events other than mutations [114]. Recently, it has been demonstrated that alternative RNA splicing of Hippo signaling regulators, including NF2 and CSNK1D, is important for their activities, and certain exon skipping of their mRNAs promotes hepatocyte proliferation and loss of mature hepatocyte functions, suggesting a novel post-transcriptional regulation of the Hippo-YAP/TAZ signaling pathway [115,116]. As well-known post-transcriptional regulators, several miRNAs have been proposed as regulators of the expression of Hippo-YAP/TAZ signaling components, and dysregulation of those miRNAs can lead to hepatic tumorigenesis [117].

In this review, we summarized the miRNAs that contribute to the development and progression of liver cancer by directly binding to the mRNAs of Hippo-YAP/TAZ signaling components or indirectly through interactions with related signaling pathways. Accumulating evidence indicates that these miRNAs could be used as biomarkers for the early detection, prognosis and monitoring of liver cancer and therapeutic targets against liver carcinogenesis. However, most of the studies have been conducted using liver cancer cell lines and xenograft models or on a relatively small number of human liver cancer specimens. Therefore, an increasing number of studies are required to investigate the functions of miRNAs in liver cancers with various etiologies and at different stages during carcinogenesis using relevant models of liver cancer and large-scale cohorts.

In conclusion, miRNAs that interact with the Hippo-YAP/TAZ signaling pathway are promising therapeutic targets for liver cancer. They play pivotal roles in hepatic tumorigenesis by affecting oncogenic transformation, proliferation and migration of tumor cells and modulating the cancer microenvironment.

## Data Availability

Not applicable.
